# Predictability limit of partially observed systems

**DOI:** 10.1038/s41598-020-77091-1

**Published:** 2020-11-24

**Authors:** Andrés Abeliuk, Zhishen Huang, Emilio Ferrara, Kristina Lerman

**Affiliations:** 1 Information Sciences Institute, University of Southern California, Marina del Rey, CA 90292 USA; 2 University of Colorado Boulder, Boulder, CO 80302 USA; 3 Department of Computer Science, University of Chile, Santiago, Chile

**Keywords:** Applied mathematics, Scientific data, Information theory and computation

## Abstract

Applications from finance to epidemiology and cyber-security require accurate forecasts of dynamic phenomena, which are often only partially observed. We demonstrate that a system’s predictability degrades as a function of temporal sampling, regardless of the adopted forecasting model. We quantify the loss of predictability due to sampling, and show that it cannot be recovered by using external signals. We validate the generality of our theoretical findings in real-world partially observed systems representing infectious disease outbreaks, online discussions, and software development projects. On a variety of prediction tasks—forecasting new infections, the popularity of topics in online discussions, or interest in cryptocurrency projects—predictability irrecoverably decays as a function of sampling, unveiling predictability limits in partially observed systems.

## Introduction

Forecasting complex dynamic phenomena—from epidemics to public opinions, stock market, and cyberattacks—is central to many policy and national security applications^1^. Accurate forecasts can help mitigate some of the risks associated with the discovery of a new software vulnerability or a viral outbreak, such as the 2019 novel coronavirus^2^. Prediction is also the standard framework in evaluating models of complex systems learned from data^3^. Time-series forecasting, which is widely used to model dynamic phenomena, represents a process as a sequence of observations (discrete or continuous counts of events) at regular time intervals. After learning parameters from past observations, the models can be used to predict future observations^4^. Forecasting models based on stochastic and self-exciting point processes, autoregressive and hidden Markov models, have been developed to predict crime^5,6^, social unrest^7^, terrorism^8^, epidemics^9^, human mobility^10^, personal correspondence^11^, online activity^12,13^, dynamics of ecological systems^14,15^ and more^16^.

A fundamental challenge to modeling efforts is the fact that complex systems are seldom fully observed. For example, when estimating opinions in a social system, it is not practical nor feasible to interview every individual in the population; instead, polling is used to elicit responses from a representative sample of a population. When social media is used as a proxy of opinions, it is similarly impractical to collect all relevant posts; instead, a (pseudo-random) sample (e.g., the Twitter *Decahose*), is often used. Further biases can emerge when data is deliberately manipulated or deleted so as to obfuscate or censor content or activity^17^. In short, the data used to learn predictive models of complex phenomena often represents a highly filtered and incomplete view.

How does data loss due to sampling affect the predictability of complex systems and the accuracy of models learned from the data? Statisticians have developed a number of approaches to compensate for data loss, including data imputation^18^ to fill in missing values, generating ensemble forecasts to account for observational uncertainty^19^ and evaluating the representativeness of sampled data^20,21^. Few of these approaches apply to temporal data. To quantify the predictability of dynamic systems, researchers use measures such as autocorrelation and permutation entropy. The former measures similarity between a time series and its own time-lagged versions. Recently, permutation entropy was introduced as a model-free, nonlinear indicator of the complexity of data^22,23^. Permutation entropy represents the complexity of a time series through statistics of its ordered sub-sequences, also known as motifs, and has been adopted to model predictability of ecological systems^14,24^ and epidemic outbreaks^9^. Despite sustained interest from researchers from very different fields, the impact of sampling and data loss on predictability of complex systems has not yet been quantitatively characterized.

As the first step towards addressing this question, we model incomplete observation as a stochastic sampling process that selects events at random with some probability *p* and drops the remaining events from observations of a system. This allows us to mathematically characterize how sampling decreases the autocorrelation of a time series. We then empirically show that sampling also reduces the predictability of a dynamic process according to both autocorrelation and permutation entropy. Moreover, the loss of predictability cannot be fully recovered from some external signal, even using data highly correlated with the original unsampled process. As a result, forecasts made by autoregressive models may be no better than predictions of simpler, less accurate models that assume independent events. We validate these findings with both synthetic and real-world data representing complex social and techno-social systems. Without any modeling assumptions on the data, we show how sampling systematically degrades the predictability of these systems.

Researchers increasingly predict complex systems and social network dynamics^1,3,25,26^ to learn the principles of human and machine behavior^27,28^. Practitioners and lawmakers alike often base their decisions on such insights^29,30^, including for public health^31,32^ and public policy^33–36^. As some pointed out^37,38^, however, caution should be used when drawing conclusions from incomplete data. Sampling, even random sampling, distorts the observed dynamics of a process, reducing its predictability. We formalize and quantify this common, yet understudied, source of bias in partially observed systems.

## Results

### Model

Consider a dynamic process generating events, for example, social media posts mentioning a particular topic, or newly infected individuals during an epidemic. We can represent the process as a time series of event counts, $X=[X_1,X_2,\ldots ,X_T]$, each entry representing the number of observations of *X* at time *t*, e.g., daily number of social media posts on a topic. We refer to this time series as the *ground truth signal*.

Observers of this process may not see all events. Twitter, for example, makes only a small fraction ($\le 10\%$) of messages posted on its platform programmatically available. Similarly, hospitals may delay reporting new cases of a disease or under-count them altogether when, for various reasons, people do not seek medical help after getting sick. We refer to the time series of observed events $Y=[Y_1,Y_2,\ldots ,Y_T]$ as the *observed signal*. Intuitively, *Y* represents a sample of events present in the ground truth signal *X*.

**Figure 1:**
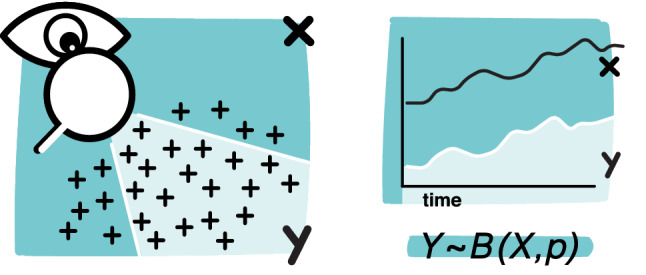
Sampling paradigm as a representation of a partially observed dynamic process. Here, *X*, the ground truth signal, represents the total events at time *t*; *Y* represents the observed subset of events. The magnifying glass illustrates the partial observability process (left) and how this distorts the observed dynamics of the process (right). The probability of an event being observed is *p*. The Binomial distribution *B*(*X*, *p*) is used to model the observed signal *Y*.

We model partial observation as a stochastic sampling process, where each event has some probability to be observed, independent of other events. This allows us to formalize how the time series of the ground truth and the observed signals are related. Figure 1 illustrates this paradigm.

#### **Definition 1**

Let *X* and *Y* be two time-series representing, respectively, the counts of total events and observed events in a dynamic system. Let sampling rate $p \in [0,1]$ be the fraction of events that are preserved by the observation process, defined by

$$\begin{aligned} Y_t \sim B(X_t,p)\;\; \forall t, \end{aligned}$$

where *B*(*n*, *p*) is a Binomial distribution with *n* trials, each with success probability *p*. Therefore, the expected number of observed events at time *t*, is ${\mathbb {E}}[Y_t] = p {\mathbb {E}}[X_t]$.

The factors driving the system may also produce some external events that may help predict the observed system. For example, rising temperatures associated with climate change may help better forecast epidemics that are made more virulent by changes in climate. Similarly, news reports may be associated with increased social media posts on specific topics, since both are driven by world events. Temperatures and news reports may provide important signals for predicting future events.

#### **Definition 2**

We define the *external signal* as a time series $S=S_1,S_2,\ldots ,S_T$ that may provide information about the ground truth signal.

### Quantifying the loss of predictability

Researchers have devised measures of predictability of complex systems. At the simplest level, autocorrelation captures how well a time series representing a complex system is correlated with its own time-lagged versions. This indicator of predictability is popular in finance^39^. In ecology and physics, permutation entropy is used to measure predictability^14,40^. Permutation entropy (PE) captures the complexity of a time series through statistics of its ordered sub-sequences, or motifs (see “Materials and methods”). The higher the permutation entropy, the more diverse the motifs, which in turn renders the time series less predictable. Permutation entropy was shown to be strongly related to Kolmogorov-Sinai (KS) entropy^41^, a theoretical measure quantifying the complexity of a dynamical system. KS is not easy to reliably estimate from data; however, for one dimensional time-series, KS and permutation entropy are known to be equivalent under a variety of conditions^22^. Using different forecasting models, Garland et al.^14^ demonstrated an empirical correlation between predictability of the models and permutation entropy^23^. Since then, PE has been used as a model-free indicator of predictability of infectious disease outbreaks^9^, human mobility^10^, ecological systems^24^, and anomaly detection in paleoclimate records^15^. Besides autocorrelation and PE, we also use prediction error as a measure of predictability^14^. However, since prediction error depends on the forecasting model, we explore it in detail only with synthetic data (SI, Synthetic Data Experiments).

We show that sampling reduces predictability of a signal, and the more data is filtered out, the less predictable the signal becomes. The loss of predictability cannot be recovered using an informative external signal, even if it is highly correlated with the original ground truth signal. We develop a framework for quantifying predictability loss due to sampling and validate it empirically using all measures of predictability.

Our main theoretical contribution is an analytical characterization of the covariance matrix of the observed signal *Y* in terms of the ground truth signal *X* and the sampling rate *p* (cf., “Materials and methods”, Theorem 1). Theorems and their proofs are presented in the SI. Based on this characterization, we derive two results stating the effects of sampling on the predictability of the observed signal *Y*:

- *Decay of autocorrelation of the observed signal.* The autocorrelation (defined as Pearson correlation between values of the signal at different times) of the observed signal *Y* decays monotonically at lower sampling rates (Corollary 2, “Materials and methods”).
- *Decay of covariance with the external signal.* The correlation between the observed and external signals degrades linearly at lower sampling rates (Corollary 3, “Materials and methods”).

Specifically, to quantify the impact of sampling on the predictability of a signal, we first derive the autocorrelation of the observed signal as a function of the sampling rate *p* (cf., Corollary 1, “Materials and methods”). When $p=1$ (i.e., complete observation), we recover the autocorrelation of the ground truth signal *X*. At lower sampling rates, the autocorrelation decays as postulated above. In parallel, we demonstrate empirically that sampling degrades predictability as measured using permutation entropy.

A forecasting model may compensate for the loss of predictability by leveraging an informative external signal. For example, auto-regressive forecasting models allow for additional covariates to improve predictions^42^. However, according to our second result, predictability cannot be fully recovered with an external signal, even one that is highly correlated with the ground truth signal.

### Empirical results

We show that sampling irreversibly degrades the predictability of real-world complex systems, studying three phenomena: disease outbreaks, online discussions, and software collaborations. Sampling reduces predictability according to both autocorrelation and permutation entropy measures, and the observed decay of autocorrelation agrees with theoretical predictions.

Predictability cannot be fully recovered using an informative external signal. In addition to co-variance, we use *mutual information* (MI) to measure the shared information between the external and the observed signals^43^. Mutual information quantifies the reduction in uncertainty about one random variable due to the presence of another^44^, and like PE it captures the non-linearities in the data that covariance cannot measure. We empirically find that sampling reduces both the covariance and MI with the external signal. The sampled time-series are obtained by stochastically sampling the ground-truth series according to Definition 1.

#### Epidemics

Scarpino and Petri^9^ used permutation entropy to show that predictability of disease outbreaks decreases over longer time periods, suggesting changes in the behavior of epidemics over time. Here, we show that the predictability of epidemics is also affected by how partially or fully observed the new infections are.

**Figure 2:**
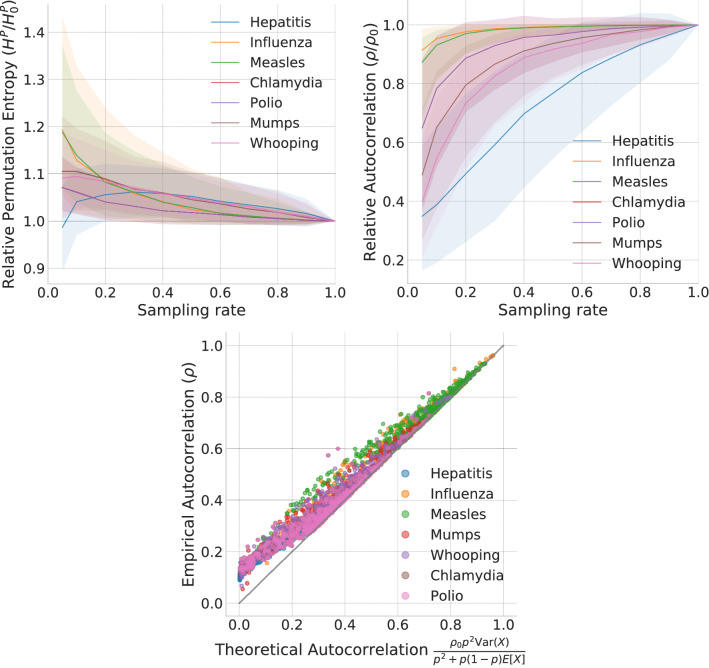
Loss of predictability of disease outbreaks due to sampling. The plots show a decrease in permutation entropy (**top-left**) and an increase in autocorrelation (**top-right**) of the outbreak time series for increasing sampling rates. For each of the eight weekly, state-level diseases, we selected 100 random 1-year time windows and calculated the relative weighted permutation entropy and autocorrelation for different sampling rates over that window. The solid line represents the median ratio across all states between the original time series and the sampled one; shaded regions mark the inter-quartile ranges. The **bottom** plot supports our theoretical results by plotting Eq. (3) against the empirical autocorrelation of the sampled time series at different sampling rates for each disease.

We study eight diseases (Chlamydia, Gonorrhea, Hepatitis A, Influenza, Measles, Mumps, Polio, and Whooping cough), representing each disease outbreak as a time series of the weekly number of reported infections in each US state. We find that at lower sampling rates, the permutation entropy (PE), over 1-year moving windows (although the results are robust to longer windows, see SI Figure 17), of the times series increases (Fig. 2 (top-left)) and the autocorrelation decreases (Fig. 2 (top-right)). Given that each disease has a different base PE and autocorrelation coefficient (see SI, Figures 14 and 15 for the absolute values), we normalized the predictability measure of the sampled time series by the corresponding measure of the ground truth time series (i.e., with full information, corresponding to sampling rate $p=1$) to capture the relative change. The observed loss of autocorrelation for each disease outbreak at different sampling rates (Fig. 2 (bottom)) agrees well with the theoretical predictions derived by Equation 3. Our findings suggest that observing only a subset of the new infections distorts the observed dynamics of the disease, making the outbreak less predictable.

**Figure 3:**
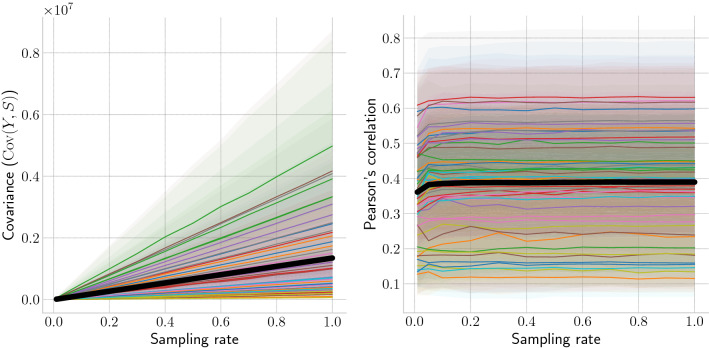
Decay of covariance between ground truth and external signals. For each state, we selected 100 random 1-year time windows and calculated the median covariance (**left**) and Pearson’s correlation (**right**) between Google Flu trends and the influenza activity at different sampling rates. Shaded regions mark the inter-quartile ranges for each state; the solid line represents the average coefficient across all states.

Next, we use influenza data to validate Corollary 3, which states that an external signal becomes less informative (i.e., has lower covariance) about the ground truth data at lower sampling rates. As an external signal *S*, we use state-level Google Flu trends^45^, which estimate influenza activity based on search queries. Figure 3 (left) shows a linear growth of covariance for each state’s influenza time series with an increasing sampling rate. However, as depicted on the right plot, there is no observed loss of correlation for lower sampling rates. This is due to the large variance relative to the mean exhibited by influenza activity. From Theorem 1, we have that the standard deviation of the observed signal *Y* is

$$\begin{aligned} \sigma _Y=\sqrt{p^2{\text {Var}}(X) + p(1-p){\mathbb {E}}[X]}\approx p\; \sigma _X \end{aligned}$$

when ${\text {Var}}(X)\gg {\mathbb {E}}[X]$. Then, it follows from Corollary 3 and the definition of Pearson’s correlation $\rho$, that

$$\begin{aligned} \rho _{Y,S}=\frac{{\text {Cov}}(Y,S)}{\sigma _Y \sigma _S} \approx \frac{p\; {\text {Cov}}(X,S)}{ p\; \sigma _X \sigma _S}=\rho _{X,S}. \end{aligned}$$

Thus, the linear decrease of covariance is offset by a linear decrease of the standard deviation. However, this is not always the case, as we later show with the cryptocurrency popularity scenario.

Supplementary Figure 16 shows that mutual information between Google Flu Trends and influenza activity also decreases, suggesting that the former becomes less informative about influenza activity the more it is sampled.

#### Social media

Next, we consider the problem of predicting social media activity. We analyze the popularity of hashtags on Twitter, defined as the daily number of posts using that hashtag. We focus on the 100 most frequently used hashtags in our data (cf., “Materials and methods”), and for each hashtag, we sample from all posts mentioning the hashtag several times at different rates to produce multiple sampled time series.

**Figure 4:**
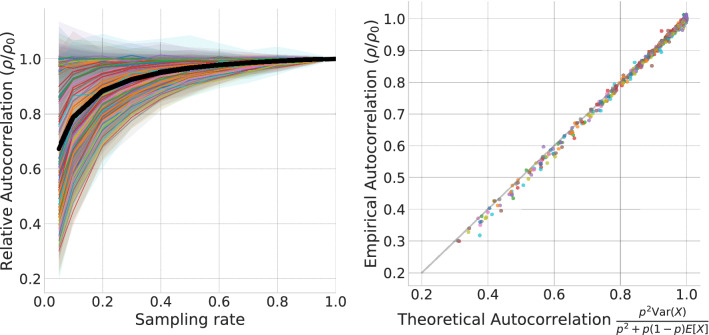
Empirical and theoretical effects of sampling on autocorrelation of hashtag popularity. (**left**) Median autocorrelation relative to the original time series for 100 most popular hashtags; shaded regions mark the inter-quartile ranges; the black line represents the average autocorrelation across all hashtags. (**right**) Accuracy of the theoretical prediction according to Eq. (3).

Figure 4 (left) shows the effects of sampling at different rates on the autocorrelation of hashtags’ popularity. The plot shows the median autocorrelation loss relative to the original time series. For each ground truth signal, we found the most significant autocorrelation time lag, which is kept fixed during the downsampling process to calculate autocorrelation at different sampling rates; then, we plotted the median ratio between the original and sampled autocorrelation. Although the curvatures are different for each hashtag, all the time series are accurately characterized by our theoretical results (Eq. 3): Fig. 4 (right) shows that the empirical loss of autocorrelation fits the theoretical predictions. Figure 21 (SI) reports the results for the sampled time series of Twitter user activity, measured by the daily number of user’s posts.

The loss of predictability is also seen when using permutation entropy with the same sampling strategy. Figures 18 and 19 (SI) show a clear trend in entropy increase (i.e., decrease of predictability) for both user activity and popularity of hashtags. The loss of predictability for user activity, for instance, happens in 63% of the users, while the rest of the cases comprise of time-series whose PE mostly do not change, except for low sampling rates (see Figure 20 (SI)).

Note that, in many applications, researchers use data from the Twitter *Decahose* or the *streaming* API, which captures approximately 10% and 1% sample of tweets, i.e., sampling rates of 0.1 and 0.01 respectively^20^. Considering that, at such low sampling rates, relative autocorrelation may be half of its value using the complete Twitter stream (*Firehose*), care should be taken when drawing conclusions from the partially observed system.

#### Cryptocurrency popularity

We present additional findings regarding the loss of correlation between a sampled time series and an external signal. We study the effect of the price of cryptocurrencies on the adoption of said technology by software developers. To measure interest in the technology behind a cryptocurrency, we track the popularity of Github projects whose description is associated with that cryptocurrency. The four most popular cryptocurrencies during the collection period spanning January 2015 to March 2015 were Bitcoin (BTC), Litecoin (LTC), Monero (XMR), and Ripple (XRP). Some cryptocurrencies, like Ethereum, were also popular, but since they were not yet publicly launched, we excluded them from the following analysis.

**Figure 5:**
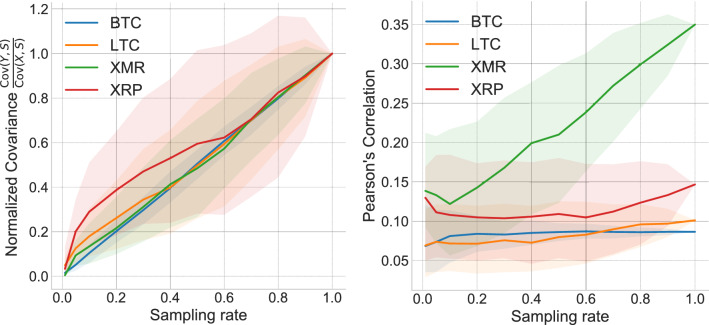
Loss of correlation between cryptocurrencies repository popularity and their prices for different sampling rates. Each point is the median Pearson’s correlation coefficient over 1000 samples. Error bars show the standard deviation. For each cryptocurrency, we calculated over the 1000 samples, (**left**) the median normalized covariance $\frac{{\text {Cov}}(Y,S)}{{\text {Cov}}(X,S)}$ and (**right**) the Pearson’s correlation coefficient between the price and the popularity of related Github repositories at different sampling rates. Shaded regions mark the inter-quartile ranges for each coin.

Figure 5 explores the effect that sampling has on the correlation. The left plot shows a clear decrease in the relative covariance for lower sampling rates, corroborating our theoretical results. As opposed to the behavior of influenza outbreaks (cf., Fig. 3), in Fig. 5 (Right) we can see that a decay of covariance tends to induce a loss of correlation, especially for those coins with low variance relative to their mean. Supplementary Figure 22 depicts a decrease in mutual information for BTC and LTC, while the other two coins are independent of the external signal.

#### Synthetic data

Finally we investigate the impact of sampling on the predictability of synthetic data generated by an auto-regressive process (SI, Synthetic Data Experiments). In addition to autocorrelation and permutation entropy, we measure the error of forecasts made by an auto-regressive model trained on the sampled data. Similar to other metrics that demonstrate a loss of predictability, prediction error grows at lower sampling rates (SI, Figure 7). As a result, the forecasts made by auto-regressive models from data collected at low sampling rates are no more accurate than forecasts made by a Poisson model that assumes independent events. Sampling further distorts the observed dynamics of the auto-regressive process by introducing heteroskedasticity into the sampled time series. The time-varying variance causes predictions to deteriorate (SI, Synthetic Data Experiments, Proposition S2).

## Materials and methods

### Sampling

#### Permutation entropy (PE)

We use permutation entropy as a model-free measure of predictability of a time series^14,23,40^. Permutation entropy captures the complexity of a time series via statistics of its ordered sub-sequences of the type $s=[x_t,x_{t+\tau },\ldots ,x_{t+(d-1)\tau } ]$, given embedding dimension *d* and a temporal delay $\tau$. Let ${\mathcal {S}}_{d,\tau }$ be the collection of all *d*! permutations $\pi$ of size *d* and temporal delay $\tau$. For each $\pi \in {\mathcal {S}}_{d,\tau }$, we determine the relative frequency $P(\pi )$ of that permutation occurring in the time series. The permutation entropy of order $d\ge 2$ and delay $\tau \ge 1$ is defined as

1 $$\begin{aligned} H^{\text {P}}(d,{\tau }) = -\sum _{\pi \in {\mathcal {S}}_{d,\tau }} P(\pi )\log _2P(\pi ) \end{aligned}$$

We use weighted permutation entropy^23^ to lessen the noise in the ordinal pattern of the signal, in which weights with respect to a sub-sequence with a certain ordinal pattern are introduced to reflect the importance of ordinal changes in large amplitudes. Finally, we *normalize* weighted permutation entropy by dividing it by $\log _2(d!)$, log of the number of possible permutations. See SI, Permutation Entropy Criterion, for a formal definition. To estimate PE of a time series we need to specify the order *d* and time delay $\tau$. The optimal parameters will depend on the specific properties of the time series, for example, the periodic behavior of the system relates to the delay parameter^46^. Here, we follow the approach described in^9^, which performs a grid search over the pairs $(d,\tau )$, $2\le d\le 5$ and $1\le \tau \le 7$ searching for the values that minimize $H^{\text {P}}(d,\tau )$. However, for the parameter search, PE is normalized by the number of observed permutations instead of the possible permutations, given that otherwise, $H^{\text {P}}(d,\tau )$ is decreasing as a function of *d*. Finally, the parameters found for each ground truth signal are used to compute the PE of the corresponding sampled time series.

#### Mutual information

Mutual information characterizes the amount of information one random variable contains about another, specifically capturing the reduction in the uncertainty of one random variable due to the knowledge of the other. The mutual information between two random variables is defined as $\displaystyle I(X;Y) = {\mathbb {E}}_{p(x,y)} \ln \frac{p(X,Y)}{p(X)p(Y)}$.

Here we consider the mutual information between two time series. We calculate the mutual information between two time series with *PyInform*^47^.

#### Loss of autocorrelation of the sampled signal

Our first theoretical result shows that sampling reduces the auto-covariance of the observed signal, i.e., the covariance of the time series *Y* and its time-lagged version.

##### **Theorem 1**

*Given the time series of event counts that assumes integer values,* $X=[X_1,\ldots ,X_T]$ *and* $Y=[Y_1,\ldots ,Y_T]$*, defined by* $Y_t \sim B(X_t,p)$*, where* *B*(*x*, *p*) *is a Bernoulli distribution with success rate* *p and* *x* *number of trials. The covariance matrices* $\varvec{\Sigma }_X$ *and* $\varvec{\Sigma }_Y$ *are related as*

2 $$\begin{aligned} \varvec{\Sigma }_Y \, \approx \, p^2\varvec{\Sigma }_X + p(1-p){\mathbb {E}}[X]{\varvec{I}}, \end{aligned}$$

*where* ${\varvec{I}}$ *is the identity matrix*.

We can use the expression in Theorem 1 to approximate the autocorrelation of the sampled time series *Y* as a function of the ground truth signal *X*. Autocorrelation is defined as Pearson correlation between values of the signal at different times, i.e., $\rho _{X_i,X_j} = \frac{{\text {Cov}}(X_i,X_j)}{\sigma _{X_i}\sigma _{X_j}}.$ For sake of simplicity, we assume that the ground truth process is stationary.

##### **Corollary 1**

*The autocorrelation of sampled time series* *Y* *is*

3 $$\begin{aligned} \rho _{Y_i,Y_j} \approx \frac{ p^2 {\text {Cov}}(X_i,X_j)}{p^2{\text {Var}}(X) + p(1-p){\mathbb {E}}[X]}. \end{aligned}$$

##### **Corollary 2**

*The magnitude of autocorrelation* $|\rho _{Y_i,Y_j}|$ *of the observed signal* *Y*, *increases monotonically as a function of the sampling rate* *p*.

##### **Corollary 3**

*The covariance between the observed signal* *Y* *and an arbitrary external signal* *S* *is related to the covariance between the ground truth signal* *X* *and the same external signal* *S* *by,*

4 $$\begin{aligned} {\text {Cov}}(Y,S) = p\,{\text {Cov}}(X,S). \end{aligned}$$

#### Epidemics data

Weekly state-level data for all diseases was obtained from Scarpino and Petri^9^ and originally compiled by the USA National Notifiable Diseases Surveillance System (see SM, Table 1 for statistics of the data). For the covariance experiment, we used influenza data from 2010 to 2015 obtained for the US Outpatient Influenza-like Illness Surveillance Network (ILINet) that overlaps with Google Flu Trends Data.

#### Twitter data

The social media data used in this study was collected from Twitter in 2014. Starting with a set of 100 users who were active discussing ballot initiatives during the 2012 California election, we expanded this set by retrieving the accounts of the users they followed, for a total of 5599 *seed users*. We collected all posts made by the seed users and their friends (i.e., users they followed on Twitter) over the period of June–November 2014, a total of over 600 thousand users. We extracted time series of the activity for 100 most popular hashtags and 150 most active users in this data (see SM, Tables 2 and 3 for statistics of the data).

#### GitHub data

The GitHub data we analyzed contains anonymized records of user activities over a time period spanning from January 1st, 2015 to March 31st, 2015. The activities represent the actions users performed on the repositories, including watching the repositories to receive notifications about project activity. We used *watches*, *forks*, and *create* event activity as a measure of popularity of a repository in Github. Overall, our dataset captures 43,962 Github activity events by 5509 users on 2036 repositories (see Supplementary Information (SI), Table 4 for additional statistics). Cryptocurrencies’ historical prices were obtained from publicly available Kaggle datasets.

## Discussion

We presented a framework to analyze the effects of partial observation of a dynamic system, identifying a fundamental limit to predictability. Using empirical data from three domains, namely epidemics, social systems, and software collaborations, we showed that data loss due to sampling degrades the predictability of disease outbreaks, social media content popularity, and the emergence of cryptocurrency technologies. We showed that even when events making up the temporal signal are sampled at random, sampling qualitatively changes the observed dynamics of the process, decreasing the autocorrelation and increasing permutation entropy. Moreover, the predictability loss is irreversible: even a highly informative external signal does not help to fully recover predictability lost to sampling. These findings were corroborated by experiments on synthetic data and empirical data.

Our work is motivated by applications requiring the forecasting of partially observed, or sampled, complex systems. Such situations may occur, for example, when country-wide forecasts of influenza have to be made based on reports by a few hospitals; when longitudinal opinion polls of a population are used to predict an election; when cyber-security models are trained on the small fraction of successful attacks^48^; or when researchers avail of random samples of social media activity to characterize complex social dynamics.

Beyond prediction, models learned from data can also elucidate social behaviors^3^. Scientists developed techniques for temporal data analysis, based on anomaly detection^49^ and regression discontinuity design^50^, to uncover natural experiments that yield insights into the mechanisms of human decision making. As we showed in this paper, however, these techniques may be systematically biased by temporal sampling. It is, therefore, imperative to account for potential sampling biases in the study of social dynamics, so that no results are erroneously attributed to the phenomena under study. Thus, it is important for future research to focus on statistical tools and sampling methods that can correct for these possible biases.

Our work suggests that partial observability not only diminishes the predictability of the inferred dynamic process based on the observations, but can also potentially mislead causal inference methods and threaten their validity. For example, interrupted time series (ITS) analyses is one of the most widely applied approaches to evaluate natural experiments in health interventions^51^. ITS consists of a sequence of counts over time, with one or more well-defined change points that correspond to the introduction of an intervention. The effect of the intervention can be estimated by fitting a linear regression model with a dummy variable for the before/after intervention, and additional variables to control for time-varying confounders. Only recently, researches have addressed methodological issues associated with ITS analysis caused by over-dispersion of time series data and autocorrelation^52^. For instance, a study estimating the impact of a ban on the offer of multi-purchase discounts by retailers in Scotland, found a 2% decrease in alcohol sales after controlling for seasonal autocorrelation, compared with a previous study’s finding no impact^53^. Our work provides a theoretical framework to understand and quantify new sources of biases due to sampling that can affect intervention studies.

## Supplementary information

Supplementary Information.

## Data Availability

This work uses publicly available data. Links to data repositories can be found in “Methods” section.

## Code availability

Codes to generate the results of the paper are available on https://github.com/aabeliuk/Predictability-partially-observed.
