# Supplementary material for: Predictability limit of partially observed systems
Source: Sci Rep. 2020 Nov 24;10:20427. doi: 10.1038/s41598-020-77091-1 (PMC7687903; doi:10.1038/s41598-020-77091-1)
Supplement: Supplementary file 1 — Supplementary Information. [file 41598_2020_77091_MOESM1_ESM.pdf]

Supplementary Information

Predictability limit of partially observed systems

Andrés Abeliuk, Zhishen Huang, Emilio Ferrara, Kristina Lerman

Proofs to Propositions and Corollaries

Proof of Theorem 1

Proof. First, we compute the off-diagonal elements of covariance matrices $\mathbf{\Sigma}_Y$ and $\mathbf{\Sigma}_X$, i.e., the relation between $\text{Cov}(X_i, X_j)$ and $\text{Cov}(Y_i, Y_j)$.

$$\begin{aligned}
\text{Cov}(Y_i, Y_j) &= \mathbb{E}[Y_i Y_j] - \mathbb{E}[Y_i]\mathbb{E}[Y_j] \\
&= \mathbb{E}_X\left[\mathbb{E}_Y[Y_i Y_j | X_i, X_j]\right] - \mathbb{E}_X\left[\mathbb{E}_Y[Y_i|X_i]\right]\mathbb{E}_X\left[\mathbb{E}_Y[Y_j|X_j]\right] \\
&= \mathbb{E}_X\left[\mathbb{E}_Y[Y_i|X_i]\mathbb{E}_Y[Y_j|X_j]\right] - \\
&\quad\ \mathbb{E}_X\left[\mathbb{E}_Y[Y_i|X_i]\right]\mathbb{E}_X\left[\mathbb{E}_Y[Y_j|X_j]\right] \\
&= \mathbb{E}_X\left[p^2 X_i X_j\right] - \mathbb{E}_X\left[pX_i\right]\mathbb{E}_X\left[pX_j\right] \\
&= p^2\big(\mathbb{E}\left[X_i X_j\right] - \mathbb{E}\left[X_i\right]\mathbb{E}\left[X_j\right]\big) \\
&= p^2 \text{Cov}(X_i, X_j) \qquad \text{(S.1)}
\end{aligned}$$

Next, we discuss diagonal elements, i.e., the relation between $\text{Var}[Y]$ and $\text{Var}[X]$. When $X_t \gg 1$, normal approximation will be used: $Y \sim B([X], p)$ is approximated by $Y \sim \mathcal{N}([X]p, [X]p(1-p))$ per Berry-Esseen theorem. Thus, *for fixed* X, $Z \equiv \dfrac{Y - pX}{\sqrt{p(1-p)X}} \sim \mathcal{N}(0, 1)$, then $Z^2 = \dfrac{Y^2 - 2pYX + p^2X^2}{p(1-p)X} \sim \chi^2(1)$, i.e.,

$$\mathbb{E}[Z^2] = \mathbb{E}\left[\frac{Y^2 - 2pYX + p^2X^2}{p(1-p)X}\right] = 1$$

This gives $\mathbb{E}[Y^2|X] = p(1-p)X + 2p\mathbb{E}[Y|X]X - p^2X^2 = p(1-p)X + p^2X^2$

$$\begin{aligned}
\text{Var}[Y] &= \text{Cov}(Y, Y) = \mathbb{E}[Y^2] - \mathbb{E}[Y]^2 \\
&= \mathbb{E}_X\left[\mathbb{E}_Y[Y^2|X]\right] - \mathbb{E}_X\left[\mathbb{E}_Y[Y|X]\right]^2 \\
&= \mathbb{E}_X[p(1-p)X + p^2X^2] - \mathbb{E}_X[pX]^2 \\
&= \mathbb{E}_X[p(1-p)X + p^2X^2] - p^2\mathbb{E}_X[X]^2 \\
&= p(1-p)\mathbb{E}[X] + p^2\text{Var}[X] \qquad \text{(S.2)}
\end{aligned}$$

Equations (S.1) and (S.2) give the desired result. □

Proof of Corollary 1

Proof. Autocorrelation is defined as Pearson correlation between values of the signal at different times, i.e.,

$$\rho_{X_i,X_j} = \frac{\mathrm{Cov}(X_i, X_j)}{\sigma_{X_i}\sigma_{X_j}}.$$

This yields the following expression for autocorrelation of the time series Y:

$$\rho_{Y_i,Y_j} = \frac{\mathrm{Cov}(Y_i, Y_j)}{\sigma_{Y_i}\sigma_{Y_j}} = \frac{p^2\mathrm{Cov}(X_i, X_j)}{\sqrt{p^2\mathrm{Var}(X_i) + p(1-p)\mathbb{E}[X_i]}\sqrt{p^2\mathrm{Var}(X_j) + p(1-p)\mathbb{E}[X_j]}}. \tag{S.3}$$

The last equality comes from replacing Equation S.1 in the numerator and Equation S.2 in the denominator. Finally, we assume the ground truth process is stationary, i.e., the process has a time-independent variance ($\mathrm{Var}(X_j)$=$\mathrm{Var}(X_j)$ $\forall i, j$) and mean ($\mathbb{E}(X_j)$=$\mathbb{E}(X_j)$ $\forall i, j$), yielding the desired result:

$$\rho_{Y_i,Y_j} \approx \frac{p^2\mathrm{Cov}(X_i, X_j)}{p^2\mathrm{Var}(X) + p(1-p)\mathbb{E}[X]}. \tag{S.4}$$

□

Proof of Corollary 2

Proof. Based on Corollary 1, the autocorrelation of the sampled signal Y between times i and j, which is a function of sampling rate p, is defined as

$$R(p) = \rho_{Y_i,Y_j} = \frac{p^2\mathrm{Cov}(X_i, X_j)}{\sqrt{p^2\mathrm{Var}(X_i) + p(1-p)\mathbb{E}[X_i]}\sqrt{p^2\mathrm{Var}(X_j) + p(1-p)\mathbb{E}[X_j]}}. \tag{S.5}$$

The autocorrelation lies in the range $[-1, 1]$, and we want to prove that its magnitude increases as a function of p. Hence, next we show that $\frac{\mathrm{d}}{\mathrm{d}p}R^2(p) \geq 0, \forall 0 \leq p \leq 1$.

$$\frac{\mathrm{d}}{\mathrm{d}p}R^2(p) = p\ \mathrm{Cov}(X_i, X_j)^2 \frac{\mathrm{Var}(X_i)\mathbb{E}[X_j]p + \mathbb{E}[X_i]\mathrm{Var}(X_j)p + 2\mathbb{E}[X_i]\mathbb{E}[X_j](1-p)}{\left(\mathrm{Var}(X_i)p - \mathbb{E}[X_i]p + \mathbb{E}[X_i]\right)^2\left(\mathrm{Var}(X_j)p - \mathbb{E}[X_j]p + \mathbb{E}[X_j]\right)^2}, \tag{S.6}$$

where both the numerator and denominator are trivially positive for all values in $0 \leq p \leq 1$ given that $\mathbb{E}[X] \geq 0$, and the result follows. □

Proof of Corollary 3

Proof. We compute the covariance between sampled signal Y and external signal S as

$$\begin{aligned}
\mathrm{Cov}(Y, S) &= \mathbb{E}(Y - \mathbb{E}Y)(S - \mathbb{E}S) \\
&= \mathbb{E}[YS] - \mathbb{E}[Y]\mathbb{E}[S] \\
&= \mathbb{E}_{X,S}\big[S\mathbb{E}_Y[Y|S, X]\big] - \mathbb{E}_X\big[\mathbb{E}_Y[Y|X]\big]\mathbb{E}[S] \\
&= \mathbb{E}_{X,S}[SXp] - \mathbb{E}_X[Xp]\mathbb{E}[S] \\
&= p\big(\mathbb{E}[SX] - \mathbb{E}[X]\mathbb{E}[S]\big) \\
&= p\,\mathrm{Cov}(X, S)
\end{aligned} \tag{S.7}$$

□

Permutation Entropy Criterion

Entropy measures the uncertainty of a random variable, which intuitively serves as an indicator of predictability of a stochastic event. In statistical physics, entropy characterizes the amount of possible microscopic state in a system, thus the more microscopic states exist in a system, the more chaotic a system is, and the harder it becomes to predict its behavior. Specifically, the Shannon entropy for a random variable $\mathbf{x}$ with distribution p is defined as $H(\mathbf{x}) = -\int p(\mathbf{x}) \ln p(\mathbf{x})\, \mathrm{d}\mathbf{x}$.

Entropy rate, on the other hand, measures the growth rate of entropy w.r.t. the length of a procession (in our setting the time series). Entropy rate of a time series X_t is defined as $\lim_{t\to\infty} \frac{H(X_1,\cdots,X_t)}{t}$, where $H(X_1, \cdots, X_t)$ is the joint entropy of random variables $X_1, \cdots, X_t$. The higher this rate is, the less predictable the time series, X_t, is. While the direct computation of such rate is difficult in practice, a quantity that approximates the entropy rate is the *permutation entropy*, which depicts the complexity of a time series through the statistics of the values of its subsequences using ordinal analysis. The complexity can be interpreted as the diversity of the trends among the subsequences of certain length (*1,2*). Therefore, the higher the permutation entropy, the more different trends exist in the time series, which renders its prediction more difficult.

Permutation entropy has been used in various fields to characterize the predictability of time series under interest (*2,3*). Interestingly, this quantity has also been used as a forensic tool to inspect and identify potential corruption in the source data (*4*).

Definition 1 (Permutation Entropy)**.** *Given a time series $\{x_t\}_{t=1}^N$. Let $\mathcal{S}_d$ be the collection of all $d!$ permutations π of order d. For each $\pi \in \mathcal{S}_d$, determine the relative frequency of that permutation occurring in $\{x_t\}_{t=1}^N$:*

$$P(\pi) = \frac{\mathrm{Card}[\{t \,|\, t \le N-d, \phi(x_{t+1}, \cdots, x_{t+d}) = \pi\}]}{N-d+1} = \sum_{t \le N-d} \frac{1}{N-d+1} \delta\big(\phi(x_{t+1}^{(d)}), \pi\big)$$

where $P(\pi)$ quantifies the probability of an ordinal pattern π, and $\delta(a, b) = \begin{cases} 1, & \text{if } a = b \\ 0, & \text{if } a \neq b \end{cases}$.
The permutation entropy of order $d \ge 2$ is defined as

$$H^{\mathrm{P}}(d) = -\sum_{\pi \in \mathcal{S}_d} P(\pi) \log_2 P(\pi) \tag{S.8}$$

The ordinal pattern means the relative magnitude relation among successive time series values. As an example, if $x_1 = 3, x_2 = 6, x_3 = 1$, then the ordinal pattern of this subsequence $\{x_1, x_2, x_3\}$ is $\phi(x_1, x_2, x_3) = (312)$ because $x_3 \le x_1 \le x_2$.

Besides the order d, the more general definition of permutation entropy (see main text Equation 1) has one more parameter: temporal delay τ. The ordinal pattern can be defined in the same way with respect to the subsequence $x_t, x_{t+1\tau}, x_{t+2\tau}, \cdots, x_{t+(d-1)\tau}$, which gives permutation entropy $H^{\mathrm{P}}(d, \tau)$.

To lessen the noise on the ordinal pattern of the signal, the *weight* w.r.t. a subsequence with certain ordinal pattern is introduced to reflect the importance of ordinal changes in large amplitude. For a subsequence of length/order d consisting times series values from x_{t+1} to x_{t+d}, which is denoted as $x_{t+1}^{(d)}$ with arithmetic mean value $\bar{x}_{t+1}^{(d)}$, its *weight* is defined (*5*) as

$$w(x_{t+1}^{(d)}) = \frac{1}{d} \sum_{j=t+1}^{t+d} (x_j - \bar{x}_{t+1}^{(d)})^2$$

As a result, the *weighted frequency of a permutation* is defined as

$$P_w(\pi) = \sum_{t \le N-d} \left(\frac{w(x_{t+1}^{(d)})}{\sum_{t' \le N-d} w(x_{t'+1}^{(d)})} \right) \delta\big(\phi(x_{t+1}^{(d)}), \pi\big)$$

The *weighted permutation entropy* is defined as

$$H_{(w)}^{\mathrm{P}}(d) = -\sum_{\pi \in \mathcal{S}_d} P_w(\pi) \log_2 P_w(\pi) \tag{S.9}$$

Here we *normalize* the weighted permutation entropy by the log-number of possible permutations, i.e., $\log(d!)$. Thus, the weighted permutation entropy takes value between 0 and 1.

Synthetic Data Experiments

Here, we validate our findings on synthetically generated time series data. We show that predictability diminishes as data is lost to sampling. We first consider an idealized scenario, where X represents an autoregressive process, from which events are sampled at random to create the observed sampled signal Y.

Synthetic Time Series Generation

External Signal First, we generate an external signal S. To assure autocorrelation, we generate it using the autoregressive integrated moving average (ARIMA) model:

$$S + \sum_{i=1}^{k} \alpha_i^{\text{ES}} S_{t-i} = \varepsilon_t + \sum_{j=1}^{l} \beta_j^{\text{ES}} \varepsilon_{t-j}. \tag{S.10}$$

ARIMA coefficients satisfy the stationarity conditions, so that the external signal S is second-order stationary. The stationarity conditions require that all roots of the polynomials $\alpha(x) = 1 + \alpha_1 x + \cdots + \alpha_k x^k$ and $\beta(x) = 1 + \beta_1 x + \cdots + b_l x^l$ satisfy $|z| > 1$; i.e., all roots of these two polynomials are located outside the unit disk. We enforce the second order stationarity by determining roots of $\alpha(x)$ and $\beta(x)$ first and then solving for the corresponding regression coefficients $\{\alpha_i\}$ and $\{\beta_j\}$, which specify the model.

Ground Truth Signal We generate the ground truth signal X in a similar manner, except that the generation model entails a term for the external signal S. This ensures that the ground truth and the external signals are correlated. Specifically, we assume the ground truth signal is defined as:

$$X + \sum_{i=1}^{K} \alpha_i^{\text{GT}} X_{t_i} = \varepsilon_t + \sum_{j=1}^{L} \beta_j^{\text{GT}} \varepsilon_{t-j} + S. \tag{S.11}$$

When generating the ground truth signal time series, we require that the *autocorrelation* of the ground truth signal is strong enough so that it can be distinguished from the random white noise.

Observed signal We sample the ground truth data X to obtain the time series of observed events, Y. The sampling rate p characterizes the probability of sampling an event. Because the count process is described by the ARIMA model, which inevitably gives real-number-valued count instead of integer-valued count, the decimal part of the count X is treated as a separate instance, and if the decision is made to keep it, its original value will be added to the posterior data. In other words, each instance of Y obeys the Binomial distribution $B(X, p)$.

Due to the stochastic nature of sampling, we generate ten different samples Y based on the ground truth signal with the same sampling rate p. In each experiment, the sampled signal Y is split into a training and testing data set, with training data used to train a predictor $\hat{Y}$ to predict the test data. The accuracy of the predictor for a given sampling rate is then averaged over the ten experiments.

Model Training Training an ARIMA model consists of two steps: First, a grid search is performed to find the best hyper-parameters (k, l), where k is the order of the autoregressive model, and l is the order of the moving-average model. For each input signal, we search over the grid for the set of parameters resulting in the lowest AIC score. Next, the corresponding coefficients of the fixed-order ARIMA model are fitted to the data.

After the best ARIMA order parameters are determined, we do step by step prediction over a specified time range. At each prediction step, the data from the previous step is incorporated into the known data as new input signal, and consequently, the model is retrained to find the updated parameters.

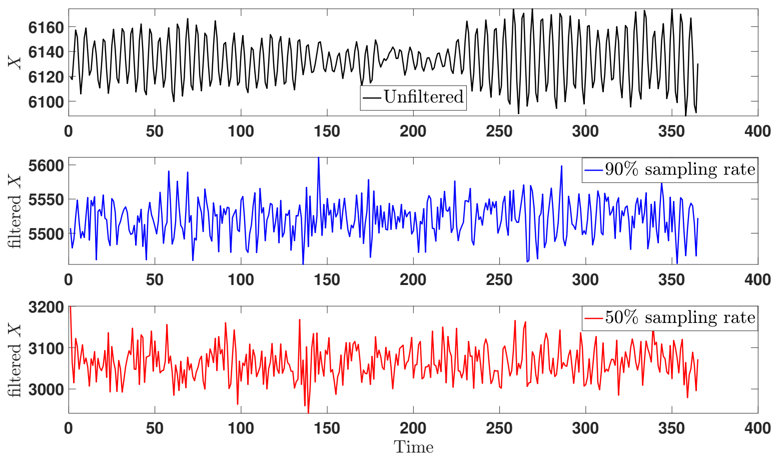

Figure S.1: Synthetic time series showing ground truth signal (top) and the observed signal sampled at sampling rates 90% (middle) and 50% (bottom).

Prediction In order to compare predictions at different sampling rates, we use normalized rooted-mean-square error (NRMSE) to measure how accurately we predict the observable Y. Given the predicted values $\hat{y}_t$ with respect to time series Y, NRMSE is defined as

$$\begin{aligned}\text{NRMSE}(y_t, \hat{y}_t) &= \frac{1}{\bar{y}}\sqrt{\frac{\sum_{i=1}^{T}(y_i - \hat{y}_i)^2}{T}} \\ &= \frac{1}{\frac{1}{T}\sum_{i=1}^{T} y_i}\sqrt{\frac{\sum_{i=1}^{T}(y_i - \hat{y}_i)^2}{T}} \qquad \text{(S.12)}\end{aligned}$$

Numeric Experiments

First, we illustrate all our theoretical claims using one instance of an ARIMA process generated with an external signal. Second, we present aggregated results of the prediction task, using multiple randomly generated ARIMA time series.

For the first set of results on the synthetic data, we generated an external signal S with the ARIMA of order (3,0,2) and length 365, representing a full year of event counts.

Meanwhile, the ground truth signal X assumes the ARIMA order(5,0,1).
Figure S.1 shows the ground truth and the sampled signals. Notice that with a simple visual inspection of the plot, one can observe that many of the temporal patterns present in the unsampled data seem to have disappeared in the sampled signal.

Predictors We train three predictors for Y, each of which can be used with or without an external signal. The predictors are:

Poisson predictor assumes that events in Y are generated independently of each other at some rate. This predictor estimates the Poisson intensity as the average of counts of all available past data.

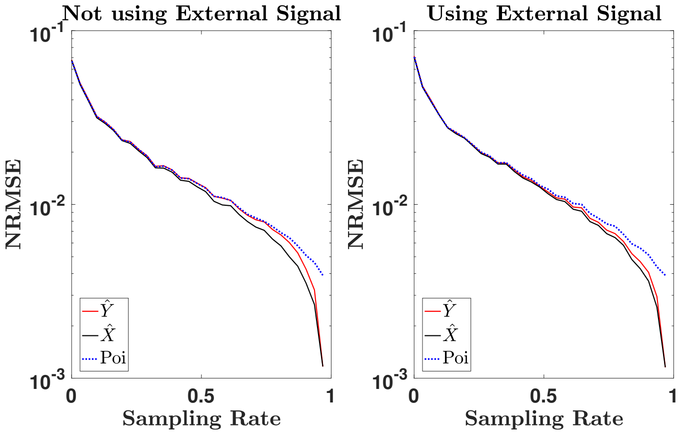

Figure S.2: Prediction accuracy in terms of NRMSE, and the difference in prediction performance among different predictors versus Sampling Rate Plot. Here we use three predictors to estimate sampled counting process Y.

$\hat{\boldsymbol{Y}}_{\boldsymbol{t}}$ uses the sampled signal to predict the observable Y.

$\hat{\boldsymbol{X}}_{\boldsymbol{t}}$ uses the unsampled signal to predict the observable Y. i.e., the ARIMA parameters are fitted to X, and then used to predict the future values of Y given its past values.

Prediction Accuracy Figure S.2 shows normalized prediction errors (normalized RMSE) as a function of sampling rate to demonstrate the nonlinear decrease of the prediction error. As sampling rate decreases, prediction error grows. We study the performance of the predictor $\hat{Y}$, which is trained on the history of the observed signal Y, as it is often employed in practice.

Performance of the predictor $\hat{X}$, trained on the full signal X, is almost always better (lower NRMSE) than performance of predictor $\hat{Y}$ (the plot show difference between predictors on the log scale). This phenomenon reveals that sampling weakens prediction accuracy. Moreover, our results suggest that the sampled process' increased noise and low autocorrelation obfuscates the underlying dynamic, making it harder to be described by an ARIMA model. Using an informative external signal S in prediction helps recover some of the lost information, shrinking the gap between $\hat{X}$ and $\hat{Y}$ predictors, as well as the overall prediction error.

When little information is lost (i.e., at high sampling rate), predictors $\hat{X}$ and $\hat{Y}$ outperform the Poisson predictor, since they are able to leverage the autocorrelation of the signal with the ARIMA model. In addition, by comparing the gaps between predictors at high sampling rates (note the log scale), we see that adding external signal makes the Poisson predictor less competitive than the other predictors. On the other hand, Poisson predictor performs almost as well as $\hat{Y}$ and $\hat{X}$ when much of the information is lost (i.e., at low sampling rate). This indicates that Poisson predictors are strong baselines for the observable Y at low sampling rates.

Loss of autocorrelation Figure S.3 shows that the autocorrelation of the sampled signal increases with sampling rate, consistent with Corollary 2. This figure shows that sampling at low rate quickly destroys the innate autocorrelation of the signal, fundamentally altering the properties of the signal and rendering the prediction task harder. We also see that the correlation between sampled ground truth signal Y and the external signal gradually increases in agreement with Corollary 3. As a consequence of the loss of

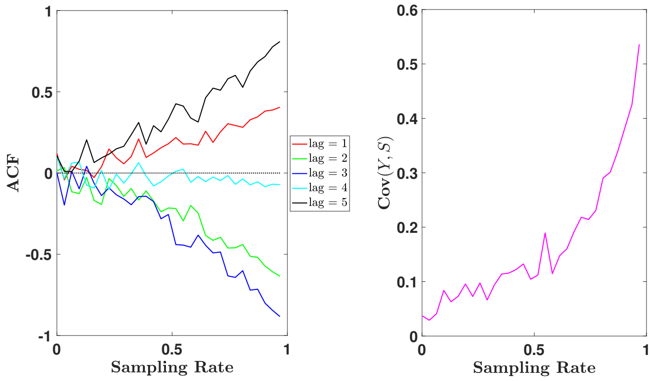

Figure S.3: Loss of predictability due to sampling. (left) autocorrelation of sampled time series decreases at low sampling rates. (right) Covariance of the external signal and observed signal decreases at low sampling rates.

autocorrelation and correlation with the external signal, we can observe in Figure S.2, that at low sampling rates, the accuracy of the Poisson predictor is competitive to the ARIMA predictors.

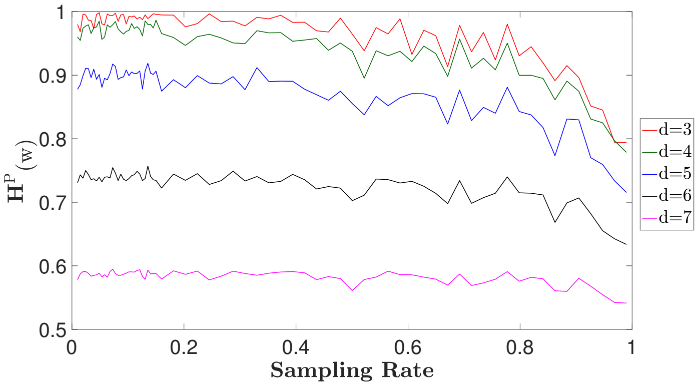

Figure S.4: Loss of predictability due to sampling for the synthetic data shown in Figure S.1. Normalized weighted permutation entropy with respect to sampling rate increases at low sampling rates, showing the system becomes less predictable. Parameter d is the embedded time dimension used to compute corresponding weighted permutation entropy $H^{\mathrm{P}}_{(\mathrm{w})}$. The delay dimension is set to $\tau = 1$.

Increase in Permutation Entropy Figure S.4 shows that the weighted permutation entropy decreases at high sampling rates, when more of the signal is retained. This shows the loss of predictability of the process at low sampling rates. The trends in the figure imply that even when little of the signal is filtered out, its predictability significantly degrades.

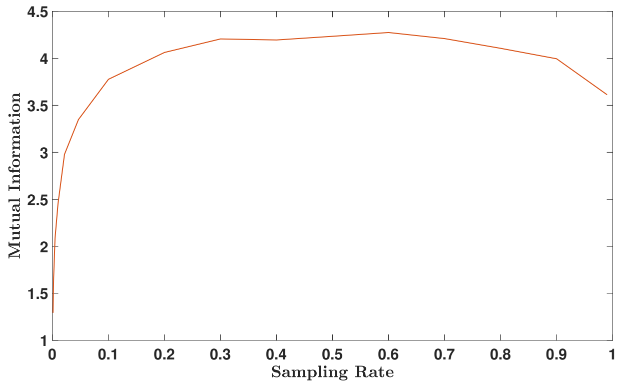

Figure S.5: Decay of mutual information between the external signal and the sampled signal. Although the external signal in this illustration is highly correlated with the original (unfiltered) ground truth signal, sampling leads to a sharp loss of information about the original signal.

Decrease in Mutual Information The loss of predictability cannot be offset using an informative external signal. This is because even if the external signal is highly correlated with the ground truth signal, sampling reduces its utility in predictive tasks. Figure S.5 shows this decay in mutual information between the external and observed signals at low sampling rates. The informative external signal does not reduce the uncertainty of the observed signal.

Nonstationarity of Prediction Errors

Another quantity that characterizes the impact of sampling on the predictability of a time series is the covariance of the prediction error at different times, as the prediction error can intuitively reflect how well one can predict the event count. We show that sampling the time-series will introduce an autocorrelation into the prediction error and render it dependent on the evolution of the counting process, with errors growing larger or smaller depending on the type of process. Next, we provide an example to motivate how sampling can induce a correlation between variances of predictions at different times.

Proposition S2. *Consider an auto-regressive (AR) process: $X_t = \alpha X_{t-1} + \varepsilon_t$, with ε_t white noise. The variance at the next step is given by,*

$$\mathrm{Var}(Y_t|X_t) = \alpha \mathrm{Var}(Y_{t-1}|X_{t-1}) + \varepsilon'_t.$$

Proof. The information filter can be described as a Binomial distribution, $Y \sim B(X, p)$. Hence, $\mathrm{Var}(Y_{t-1}|X_{t-1}) = X_{t-1}p(1-p)$ using the fact that $Y_{t-1}|X_{t-1}$ is a Bernoulli random variable. Then, the variance at the next step is given by,

$$\begin{aligned}\mathrm{Var}(Y_t|X_t) =& X_t p(1-p) \\ =& \left(\alpha X_{t-1} + \varepsilon_t\right) p(1-p) \\ =& \alpha X_{t-1} p(1-p) + \varepsilon'_t \\ =& \alpha \mathrm{Var}(Y_{t-1}|X_{t-1}) + \varepsilon'_t.\end{aligned}$$

□

Proposition S2 shows that the variances of the sampled process, Y, are related by the exact same AR model that generated the process X. In other words, the conditional variance of the sampled process is

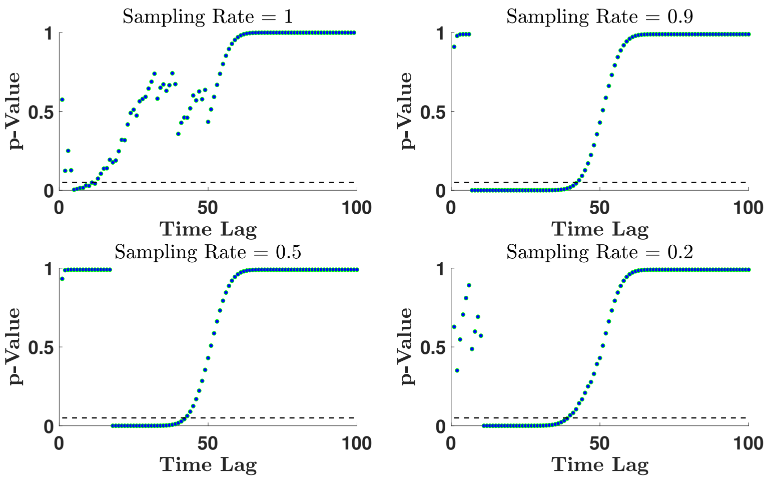

Figure S.6: p-Value versus time lag

autocorrelated. Notice, that this is not true for the unsampled data, given that $\mathrm{Var}(X_t|X_{t-1}) = \mathrm{Var}(\varepsilon_t) = \sigma^2$.

Proposition S2 shows that sampling a time series may introduce autoregressive conditional heteroskedasticity (ARCH) of the variance into the time series. This is usually tested by analyzing the residuals of the model. We use Engle's Langrage Multiplier test to demonstrate the appearance of ARCH effects in the residual signal. Figure S.6 shows that sampling does result in the introduction of ARCH effects to the residuals of predictions.

We have applied Engle's Lagrange Multiplier (ELM) test (*6*) on the residual $\hat{Y} - Y$, which is evaluated at time $t, t-1, \cdots, t-L$ where $L = 100$ is the maximum time lag we consider, to examine the existence of ARCH behavior. Under the null hypothesis that there is no ARCH effect, the test statistic used in ELM test has the asymptotic distribution $\chi^2(L)$. When p-value is less than the significance level $\tilde{\alpha} = 0.05$, the ELM test says that we can reject the null hypothesis with 95% confidence.

From Figure S.6, we see that for the original time-series (top-left plot) there is no heteroskedasticity, p-values are largely over the significant level threshold 0.05 for most of time lags under consideration. In the case of $q = 0$, ELM test does not provide us enough evidence to reject the hypothesis that ARCH effect does not exist. However, when we filter the original signal, as we see in the case $q = 0.1, 0.5, 0.8$ respectively, the p-value for time lags from 6 to 40 are mostly below the significance level bar, which, by ELM test, strongly suggests that the null hypothesis be rejected. In other words, ELM test suggests with 95% confidence that ARCH effect exists in the residual of prediction records on the filtered time-series.

Generalizability

So far, we have explored methodically one example where we have validated our theoretical results as well as provided new insights about the unpredictability of sampled time series. Here we show the average loss of predictability across many randomly generated ground truth time series.

We generate multiple external signal–ground truth signal pairs, and apply ARIMA and Poisson models on these data sets to test if the tendencies we have observed previously (i.e. nonlinear decrease of NRMSE with respect to sampling rate) will also appear in signals of different ARIMA orders. We observe in Figure S.7 similar behavior across multiple data sets:

1. Increasing prediction error (NMRSE) despite small discrepancy at high sampling rates. This gen-

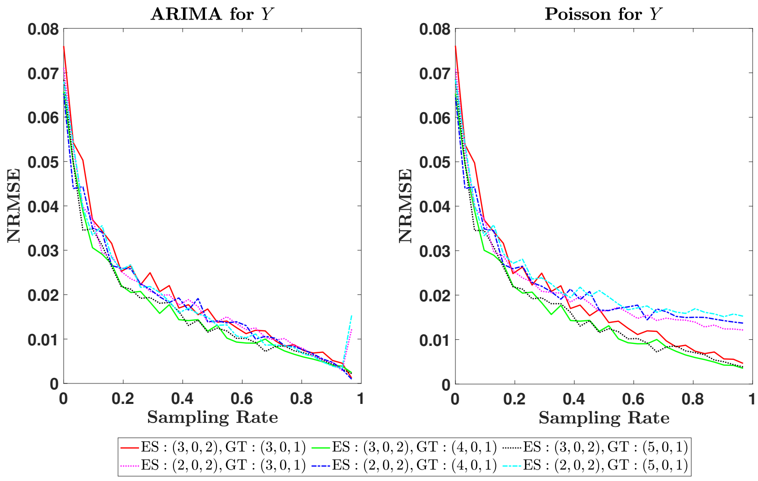

Figure S.7: Change of prediction accuracy (RMSE) with respect to sampling rate for various ground truth signals, when the sampled GTs are predicted without knowing external signal S.

eral behavior is partially due to the normalization by sample mean when we evaluate the NRMSE;

2. The autoregressive model outperforms the non-autoregressive Poisson model for higher sampling rates, but, this difference fades out for lower sampling rates as a consequence of sampling.

We further postulate that this decreasing tendency between increasing sampling rate and NRMSE bears generality with respect to any combination of ARIMA orders of external signal–ground truth data pair. When predicting Y with the external signal S, we observe a very similar tendency of NRMSE—sampling rate relationship as in Figure S.7. Albeit noticeable difference in the high end of sampling rate spectrum, the decreasing tendency, decreasing speed and the amplitude of NRMSE are by large the same as in the not-using-S case.

In the general setting of ARIMA model, we show a decreasing prediction error w.r.t. the sampling rate, and the shrinking of the advantage of autoregressive model in prediction accuracy over Poisson model as sampling rate becomes smaller.

We demonstrated the decoupling of the external signal and the filtered signal as a prevalent phenomenon in the generic ARIMA setting. We observe in Figure S.8 that for all ground truth-external signal pairs, the lower the sampling rate is, the smaller mutual information becomes. Therefore, we postulate that the positive correlation between sampling rate and mutual information can always be observed, thus implying that the knowledge of external signal cannot help recover the predictability of the ground truth signal.

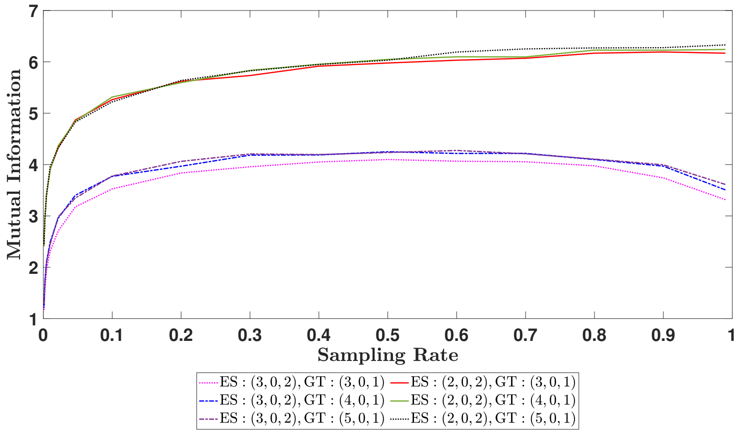

Figure S.8: Change of mutual information (MI) with respect to sampling rate for various (ground truth, external signal) pairs.

Supplementary Figures

Epidemics.

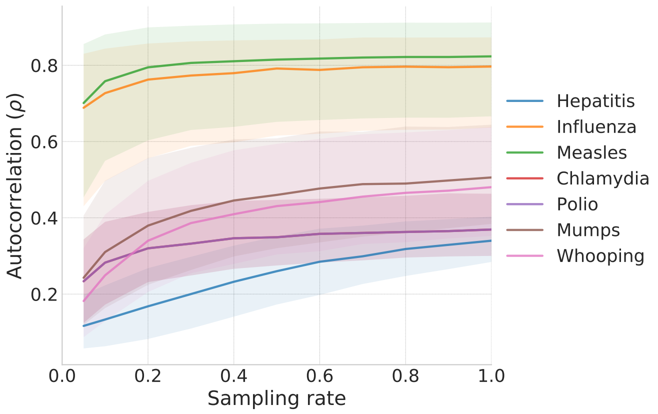

Figure S.9: Theoretical and empirical Loss of autocorrelation in outbreaks due to sampling for all diseases. The plot depicts a decrease on the autocorrelation as drop-out rate increase. For each of the eight weekly, state-level diseases, we selected 100 random points and calculated the entropy and autocorrelation for different drop-out rates over a one year window. The solid line represents the median, shaded region marks the interquartile range.

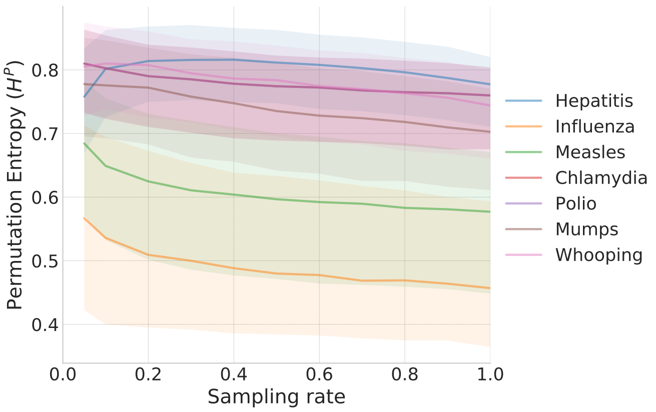

Figure S.10: Loss of predictability due to sampling for all diseases. The plot shows and increase on the permutation entropy as drop-out rate increase. For each of the eight weekly, state-level diseases, we selected 100 random points and calculated the entropy and autocorrelation for different drop-out rates over a one year window. The solid line represents the median, shaded region marks the interquartile range.

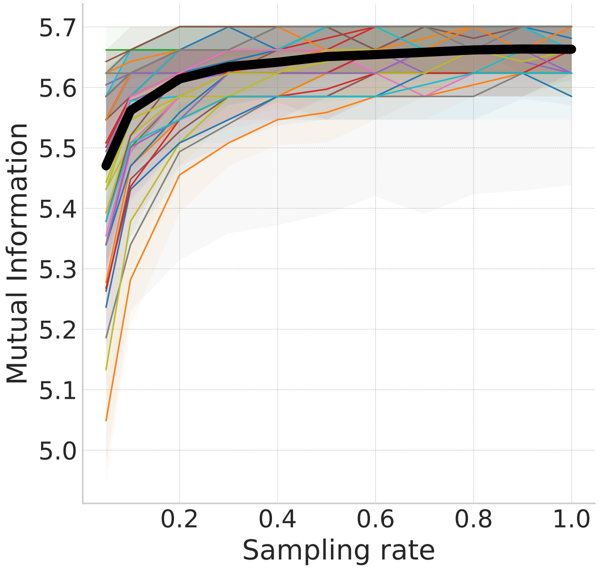

Figure S.11: Decay of mutual information with external signal due to sampling of the influenza activity. For each state, we selected 100 random one-year time windows and calculated the median mutual information (*7*) between Google Flu trends and the influenza activity at different sampling rates. Shaded regions mark the inter-quartile ranges for each state; the solid line represents the average coefficient across all states.

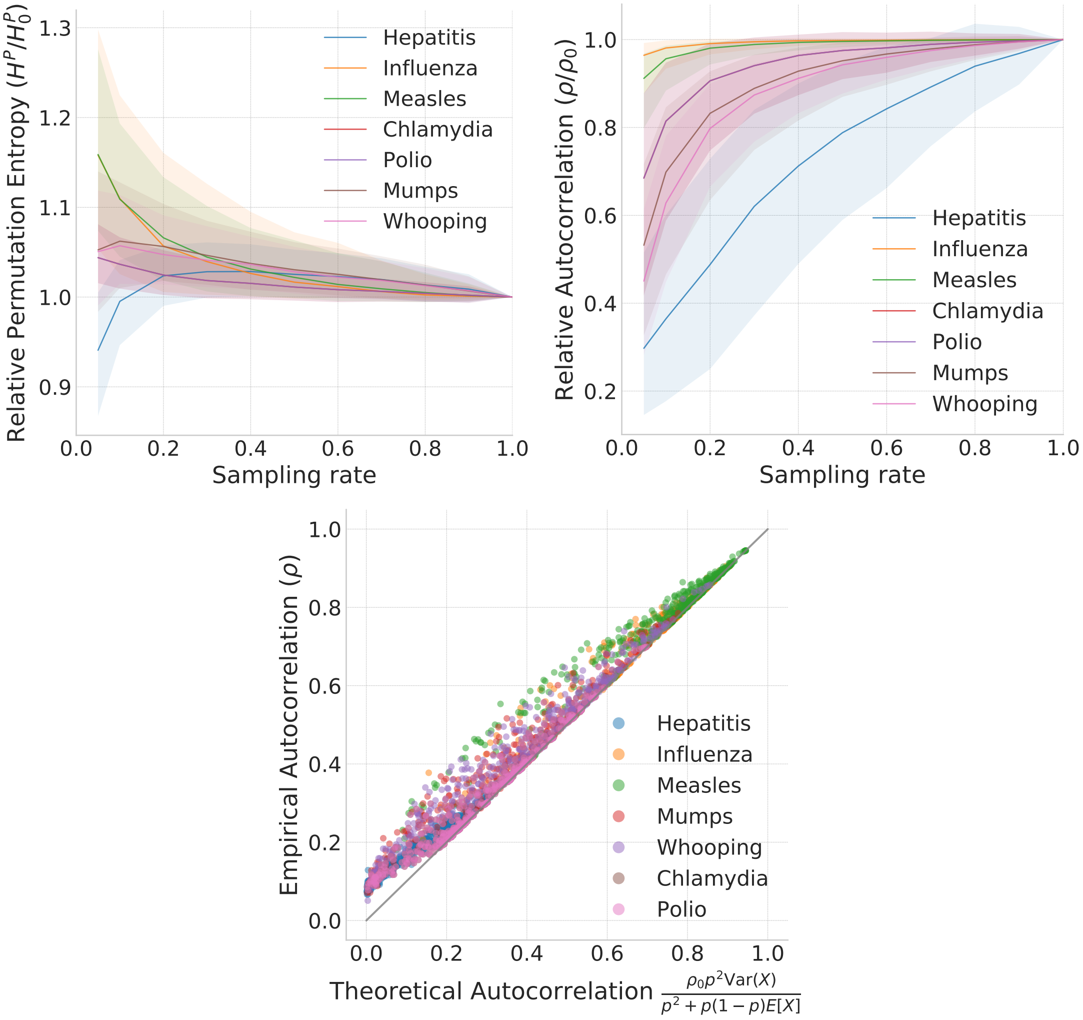

Figure S.12: Loss of predictability of disease outbreaks due to sampling. The plots show a decrease in permutation entropy (**top-left**) and an increase in autocorrelation (**top-right**) of the outbreak time series for increasing sampling rates. For each of the eight diseases, we selected 100 random two-year time windows and calculated the relative weighted permutation entropy and autocorrelation for different sampling rates over that window. The solid line represents the median ratio across all states between the original time series and the sampled one; shaded regions mark the inter-quartile ranges. The **bottom** plot supports our theoretical results by plotting Equation 3 against the empirical autocorrelation of the sampled time series at different sampling rates for each disease.

Social Media.

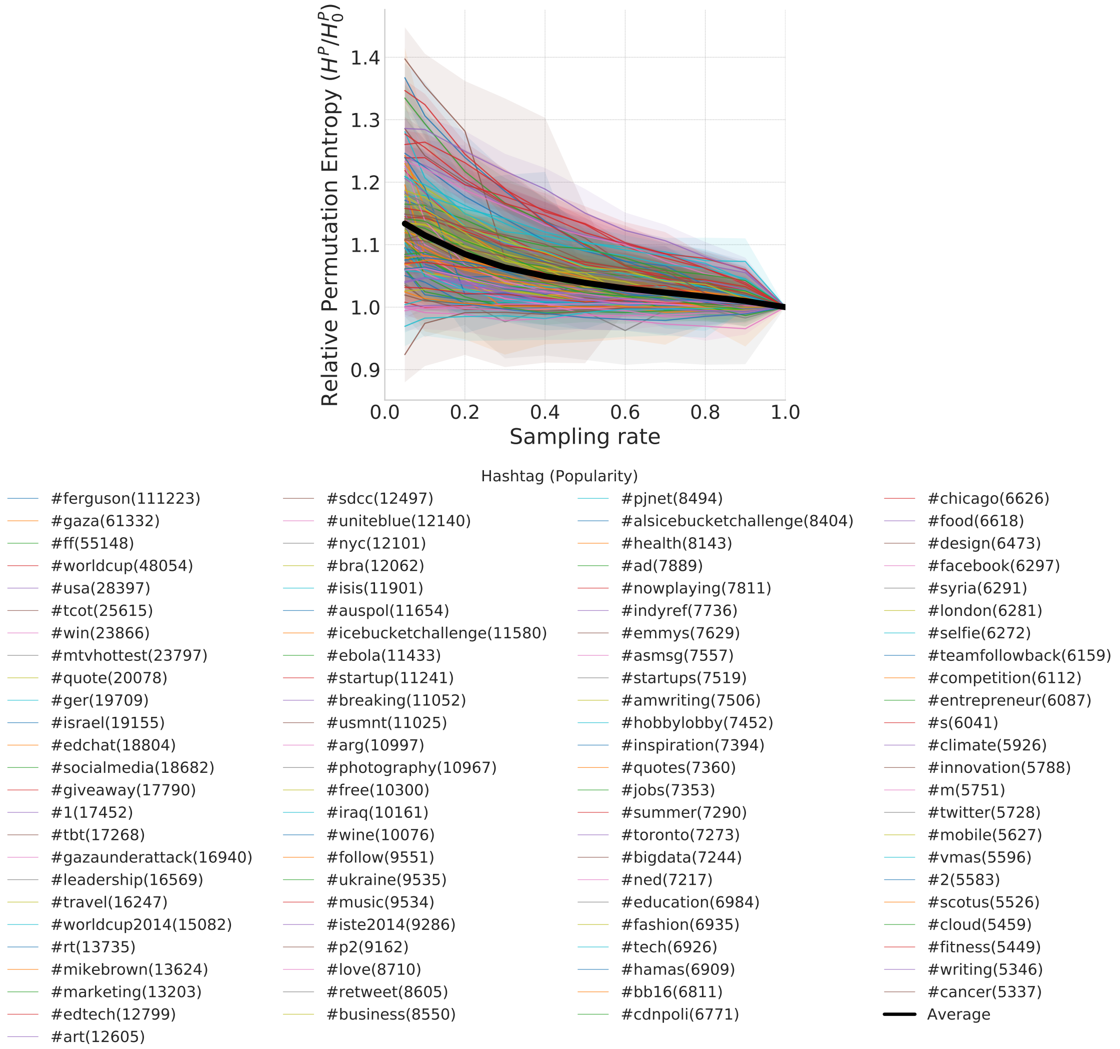

Figure S.13: Loss of predictability in social media due to sampling on user's activity (posts made by the user). The plot shows the median weighted permutation entropy relative to the original time series for each of the top 100 most popular hashtags.

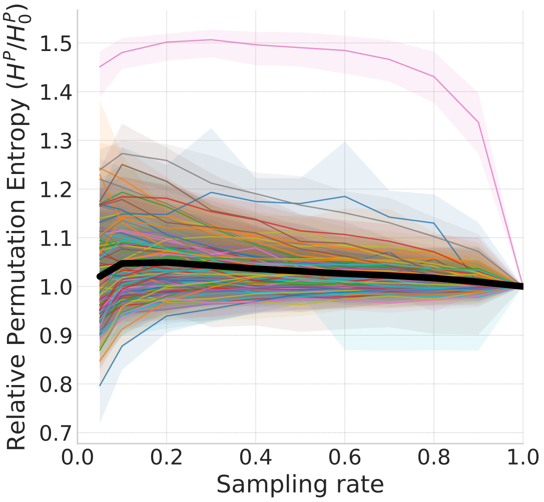

Figure S.14: Loss of predictability in social media due to sampling on user's activity (posts made by the user). The plot shows the median weighted permutation entropy relative to the original time series for each of the top 100 most active users.

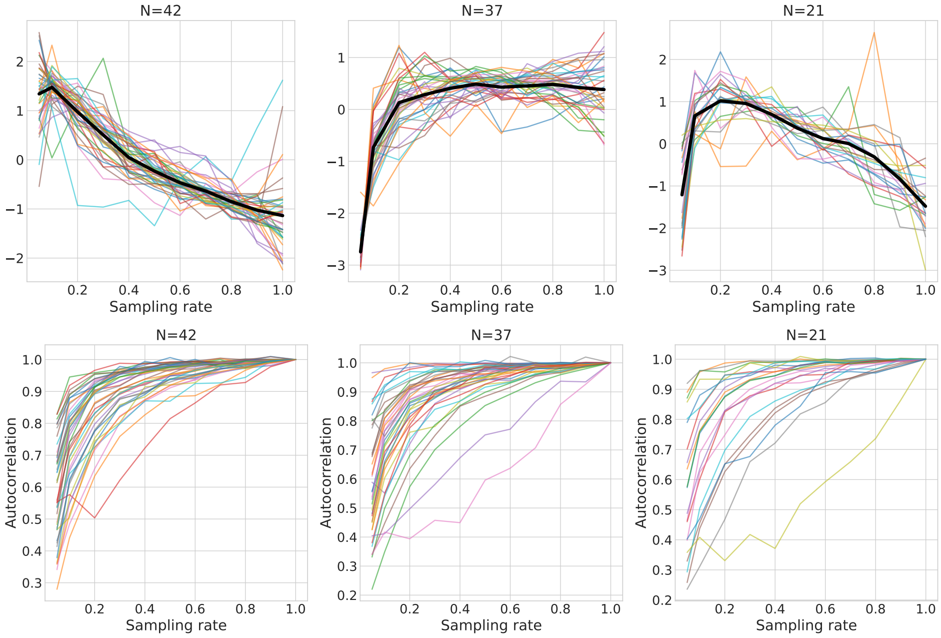

Figure S.15: Clustering the permutation entropy behavior from Figure S.14. We use, K-means clustering to depict the three most characteristics types of behaviors exhibited when computing predictability in social media as a function of sampling on user's activity.

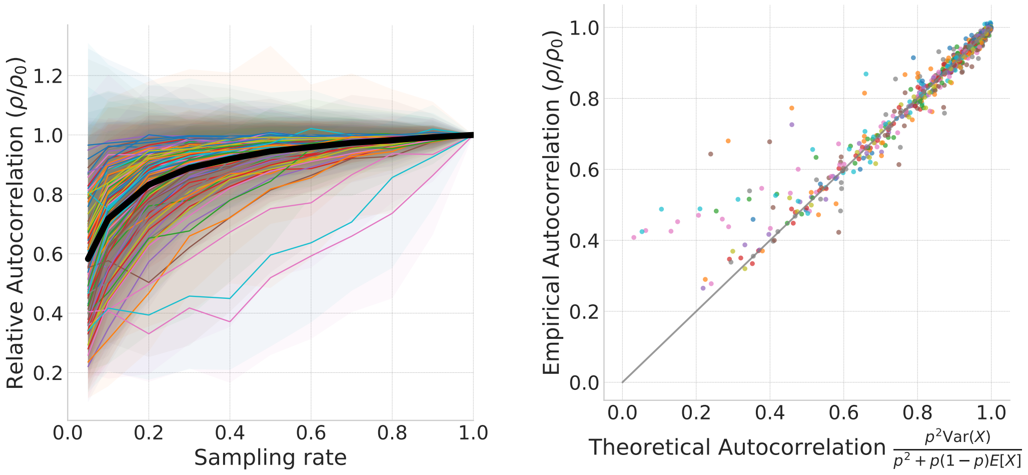

Figure S.16: Empirical and theoretical effects of sampling on autocorrelation of user's activity. The left plot shows the median autocorrelation relative to the original time series for each of the top 100 most active users; shaded region marks the interquartile ranges; the black line represents the average autocorrelation across all users. The right plot shows the accuracy of the theoretical prediction according to Equation 3. The line depicts the identity function to represent an accurate fit to the data.

Cryptocurrencies.

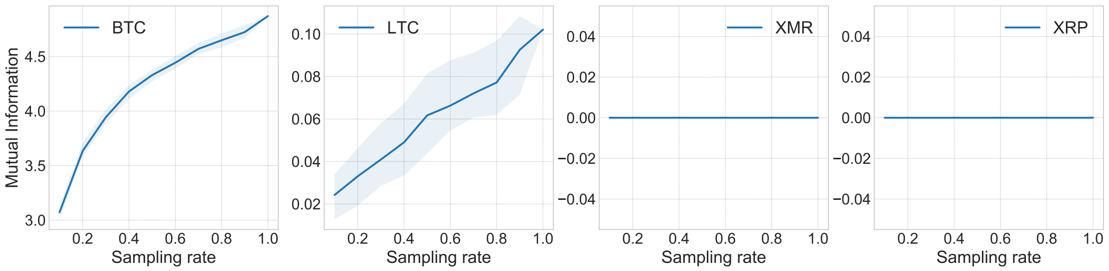

Figure S.17: Decay of mutual information between the popularity of cryptocurrencies repositories and their prices for different sampling rates. For each cryptocurrency, and each sampling rate, we obtained 100 samples, and calculated the median mutual information (7) between the price and the popularity of related Github repositories. The solid line represents the median mutual information for each coin. Shaded region marks the interquartile ranges for each coin.

Supplementary Tables

Disease	Years	Total Infections	Infections/Year (SD)	Infections/week (SD)
Hepatitis	1966-2014	$742,554$	$15,154$ $(18,905)$	$14,011$ $(2,643)$
Influenza	1919-1951	$6,498,817$	$196,934$ $(198,551)$	$122,619$ $(163,348)$
Measles	1909-2001	$18,430,036$	$198,172$ $(247,833)$	$347,737$ $(310,744)$
Chlamydia	2006-2014	$4,882,110$	$542,456$ $(80,216)$	$92,115$ $(18,487)$
Polio	1921-1971	$505,246$	$9,907$ $(13,134)$	$9,532$ $(10,676)$
Gonorrhea	1972-2014	$3,701,913$	$86,091$ $(210,930)$	$69,847$ $(13,426)$
Mumps	1967-2014	$866,965$	$18,061$ $(35,054)$	$16,357$ $(9,180)$
Whooping cough	1909-2014	$2,220,008$	$20,943$ $(47,835)$	$41,887$ $(7,296)$

Table S.1: Epidemics data: descriptive statistics. For each disease, we report the years of data recollected; the total number of infections; average infections per year; average infections per week; and the standard deviations (SD).

Hashtags	Users	Tweets	Tweets/hashtag (SD)	Tweets/User (SD)	Tweets/Day (SD)
100	$233,108$	$1,269,348$	$12,693$ $(13,561)$	5.4 (12)	$11,135$ $(6,941)$

Table S.2: Twitter hashtag activity descriptive statistics. Top 100 most popular hashtags. We report the total number of users mentioning the 100 most popular hashtags; the total number of Tweets containing these hashtags; the average number of Tweets per hashtag; average number Tweets per user; average number Tweets per day; and the standard deviations (SD).

Users	Tweets	Hashtags	Tweets/User (SD)	Tweets/hashtag (SD)	Tweets/Day (SD)
150	$167,654$	$20,990$	$1,118$ $(13,561)$	8.0 (41.6)	$1,552$ $(1,668)$

Table S.3: Twitter user activity descriptive statistics. Top 150 most active users. We report the total number of Tweets by the 150 most active users; the total number of Hashtags by these users; the average number of Tweets per user; the average number of Tweets per hashtag; the average number of Tweets per day; and the standard deviations (SD).

Cryptocurrency	Events	Repositories	Users	Events/Day (SD)	Events/Repo (SD)
Bitcoin (BTC)	$40,038$	$1,962$	$5,324$	460 (116)	20.4 (138.4)
Ripple (XRP)	$2,963$	7	86	35.3 (27.7)	423.2 $(1,115)$
Litecoin (LTC)	$1,222$	137	302	14 (11)	8.9 (19.2)
Monero (XMR)	370	15	54	5.7 (6.3)	24.7 (55.8)

Table S.4: Github data: descriptive statistics. For each cryptocurrency, we report the total number of activity events (*watches*,*forks*, and *create*); the total number of repositories where these events happened; the total number of users responsible for the activity; the average number of events per day; the average number of events per repository; and the standard deviations (SD).

References

1. K. E. Petersen, *Ergodic Theory*, Cambridge Studies in Advanced Mathematics (Cambridge University Press, 1983).
2. J. Garland, R. James, E. Bradley, *Physical Review E* **90**, 052910 (2014).
3. A. Politi, *Physical review letters* **118**, 144101 (2017).
4. J. Garland, *et al.*, *Entropy* **20**, 931 (2018).
5. B. Fadlallah, B. Chen, A. Keil, J. Príncipe, *Physical Review E* **87**, 022911 (2013).
6. R. F. Engle, *Econometrica: Journal of the Econometric Society* pp. 987–1007 (1982).
7. D. G. Moore, G. Valentini, S. I. Walker, M. Levin, *Frontiers in Robotics and AI* **5**, 60 (2018).
